# In vitro antifungal activity of hydroxychavicol isolated from *Piper betle *L

**DOI:** 10.1186/1476-0711-9-7

**Published:** 2010-02-03

**Authors:** Intzar Ali, Farrah G Khan, Krishan A Suri, Bishan D Gupta, Naresh K Satti, Prabhu Dutt, Farhat Afrin, Ghulam N Qazi, Inshad A Khan

**Affiliations:** 1 Clinical Microbiology Division Indian Institute of Integrative Medicine, Canal Road, Jammu-180 001, India; 2 Natural Product Chemistry Division, Indian Institute of Integrative Medicine, Canal Road, Jammu-180 001, India; 3 Department of Microbiology, Acharya Shri Chander College of Medical Sciences, Sidhra, Jammu-180 017, India; 4 Department of Biotechnology, Faculty of Science, Hamdard University, Hamdard Nagar, New Delhi-110 062, India

## Abstract

**Background:**

Hydroxychavicol, isolated from the chloroform extraction of the aqueous leaf extract of *Piper betle *L., (Piperaceae) was investigated for its antifungal activity against 124 strains of selected fungi. The leaves of this plant have been long in use tropical countries for the preparation of traditional herbal remedies.

**Methods:**

The minimum inhibitory concentration (MIC) and minimum fungicidal concentration (MFC) of hydroxychavicol were determined by using broth microdilution method following CLSI guidelines. Time kill curve studies, post-antifungal effects and mutation prevention concentrations were determined against *Candida *species and *Aspergillus *species "respectively". Hydroxychavicol was also tested for its potential to inhibit and reduce the formation of *Candida albicans *biofilms. The membrane permeability was measured by the uptake of propidium iodide.

**Results:**

Hydroxychavicol exhibited inhibitory effect on fungal species of clinical significance, with the MICs ranging from 15.62 to 500 μg/ml for yeasts, 125 to 500 μg/ml for *Aspergillus *species, and 7.81 to 62.5 μg/ml for dermatophytes where as the MFCs were found to be similar or two fold greater than the MICs. There was concentration-dependent killing of *Candida albicans *and *Candida glabrata *up to 8 × MIC. Hydroxychavicol also exhibited an extended post antifungal effect of 6.25 to 8.70 h at 4 × MIC for *Candida *species and suppressed the emergence of mutants of the fungal species tested at 2 × to 8 × MIC concentration. Furthermore, it also inhibited the growth of biofilm generated by *C. albicans *and reduced the preformed biofilms. There was increased uptake of propidium iodide by *C. albicans *cells when exposed to hydroxychavicol thus indicating that the membrane disruption could be the probable mode of action of hydroxychavicol.

**Conclusions:**

The antifungal activity exhibited by this compound warrants its use as an antifungal agent particularly for treating topical infections, as well as gargle mouthwash against oral *Candida *infections.

## Background

Mycosis constitutes a common health problem, especially in tropical and subtropical developing countries; dermatophytes, *Malassezia *species and *Candida *species being the most frequent pathogens in humans and animals [1]. In recent years, there has been an increasing search for new antifungal agents. However, since many of the available antifungal drugs have undesirable side effects or are very toxic (amphotericin B), produce recurrence, show drug-drug interactions (azoles) or lead to the development of resistance (fluconazole, 5-flucytosine), some shows ineffectiveness [2,3] and have become therefore less successful in therapeutic strategies. Therefore it is necessary to search for more effective and less toxic novel antifungal agents that would overcome these disadvantages. Interestingly, plants are widely employed in folk medicine, mainly in communities with inadequate conditions of public health and sanitation. Several medicinal plants have been extensively studied in order to find more effective and less toxic compounds [4].

*Piper betle *L., (Piperaceae) has been extensively used in traditional herbal remedies in India, China, Taiwan, Thailand and many other countries. It is reported for various pharmacological activities such as antimicrobial, antioxidant, antimutagenic, anticarcinogenic, antiinflammatory [5] etc. It also acts as a stimulant, a breath freshener, a carminative, a sialagogue, a cardiac tonic, a pain killer in joint pain, an aphrodisiac, an astringent, an antiseptic [5-7], a digestive and pancreatic lipase stimulant [8], wound healing [9].

Hydroxychavicol is the major phenolic component, isolated from the aqueous extract of *P. betle *L., leaf has been reported to possess antinitrosation, antimutagenic, anticarcinogenic activities [10]. It also has a tendency to act as an antioxidant, and a chemopreventive agent [10]. Other useful properties include antiinflammatory, antiplatelet and antithrombotic without impairing haemostatic functions [11]. There have been reports on the antibacterial activities of hydroxychavicol [12,13], but so far the report on its antifungal activity is lacking.

The present study was sought to investigate the effects of hydroxychavicol on fungal pathogens. In addition its effect on membrane permeability of *C. albicans *was also examined.

## Methods

### Antifungal agents

**Figure 1:**
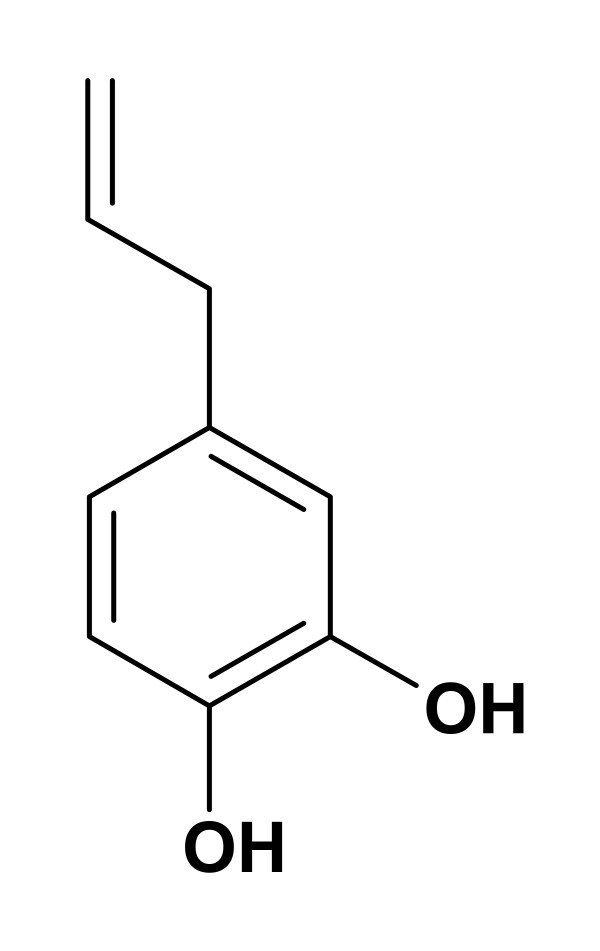
**Structure of hydroxychavicol**.

Hydroxychavicol (Fig. 1) was isolated in the pure form from the chloroform extraction of the aqueous leaf extract of *P. betle *L., (Piperaceae) as described previously [12]. Amphotericin B was purchased from Sigma Chemical Co. (St. Louis, MO), and terbinafine was obtained as kind gift from Lupin Laboratories, Pune, India.

### Fluorochrome dye

Propidium iodide (Sigma), a small cationic, nucleic acid-binding fluorochrome largely excluded by intact cell membranes was used to stain the yeast cells [14]. Sodium deoxycholate (Sigma), an anionic detergent, was used to facilitate diffusion of propidium iodide into the yeast cell membranes which were damaged by the antifungal agent [15].

### Fungal strains and growth conditions

A total of 124 fungal strains were tested for their susceptibility to hydroxychavicol. These strains comprised of *Candida albicans *(ATCC 90028, ATCC 10231 and 23 clinical isolates), *Candida glabrata *(ATCC 90030 and 7 clinical isolates), *Candida krusei *(ATCC 6258 and 3 clinical isolates), *Candida parapsilosis *(ATCC 22019 and 5 clinical isolates), *Candida tropicalis *(ATCC 750 and 11 clinical isolates), *Cryptococcus neoformans *(ATCC 204092 and 2 clinical isolates), *Aspergillus flavus *(MTCC 1973, MTCC 2799 and 10 clinical isolates), *Aspergillus fumigatus *(MTCC 1811 and 17 clinical isolates), *Aspergillus niger *(ATCC 16404 and 6 clinical isolates), *Aspergillus parasiticus *(MTCC 2796), *Epidermophyton floccosum *(MTCC 613 and 1 clinical isolate), *Microsporum canis *(MTCC 2820 and 3 clinical isolates), *Microsporum gypsium *(MTCC 2819 and 2 clinical isolates), *Trichophyton mentagrophytes *(ATCC 9533 and 7 clinical isolates), and *Trichophyton rubrum *(MTCC 296 and 9 clinical isolates). Reference strains were procured from the American Type Culture Collection (ATCC, Manassas, VA, USA), and Microbial Type Culture Collection (MTCC, Chandigarh, India). The clinical isolates were obtained from the Department of Microbiology, Acharya Shri Chander College of Medical Sciences, Sidhra, Jammu, India.

### "Inoculum" preparation

Suspensions of the yeasts and *Aspergillus *species were prepared in sterile normal saline (0.85%) containing 0.05% polysorbate 20 (NST) from 24 h (48 h for *C. neoformans*) and 7-day-old cultures "respectively" grown on potato dextrose agar (Difco Laboratories, Detroit, Mich) at 35°C [16,17]. A stock inoculums suspension of each dermatophytes was prepared from fresh, mature (7-day-old) cultures grown on potato dextrose agar with 2% in-house rice flour slants at 28°C. The densities of these suspensions were adjusted with a spectrophotometer (Multiskan spectrum, Thermo electron, Vantaa, Finland) at a wavelength of 530 nm to a transmittance of 65 to 70% to yield an initial inoculum of 1 × 10^6 ^to 5 × 10^6 ^cfu/ml [18]. All adjusted suspensions were quantified by plating on Sabouraud dextrose agar (SDA; Difco Laboratories) plates.

### MIC and MFC determination of hydroxychavicol

The MIC was performed by broth microdilution methods as per the guidelines of Clinical and Laboratory Standard Institute (formerly, the National Committee for Clinical Laboratory Standards) [16,17], with RPMI 1640 medium containing L-glutamine, without sodium bicarbonate and buffered to pH 7.0 with 0.165 M morpholinepropanesulfonic acid (RPMI) (both from Sigma). Stock solution of hydroxychavicol was prepared in 100% dimethyl sulfoxide (DMSO; Sigma) and twofold serial dilutions were prepared in media in amounts of 100 μl per well in 96-well U-bottom microtiter plates (Tarson, Mumbai, India). The above-mentioned fungal suspensions were further diluted in media, and a 100 μl volume of this diluted inoculum was added to each well of the plate, resulting in a final inoculum of 0.5 × 10^4 ^to 2.5 × 10^4 ^cfu/ml [19] for yeasts and 0.4 × 10^4 ^to 5 × 10^4 ^cfu/ml for dermatophytes and *Aspergillus *species. The final concentration of hydroxychavicol ranged from 3.90 to 2000 μg/ml. The medium without the agents was used as a growth control and the blank control used contained only the medium. Amphotericin B and terbinafine served as the standard drug controls. The microtiter plates were incubated at 28°C for 7 days for dermatophytes [18], and at 35°C for 48 h for *Candida *species (72 h for *C. neoformans*) and *Aspergillus *species [16,17]. The plates were read visually, and the MIC was defined as the lowest concentration of the antifungal agents that prevented visible growth with respect to the growth control.

The MFC was determined by plating a 100 μl volume on SDA from the wells showing no visible growth. The plates were incubated as described above in MIC. The minimum concentration of hydroxychavicol that showed ≥ 99.9% reduction of the original inoculums was recorded as the MFC [19].

### Time kill curve studies

Time-kill curve studies were performed as described by Ernst et al [20], using RPMI. *C. albicans *ATCC 90028 and *C. glabrata *ATCC 90030 were used as the test strains in this study. One milliliter of the adjusted inoculum suspension (≈ 5 × 10^6 ^cfu/ml) was added to nine ml of RPMI with or without hydroxychavicol, providing the starting inoculum of ≈ 5 × 10^5 ^cfu/ml. The range of hydroxychavicol concentrations tested was one to eight times the MICs for test strains i.e. 250 to 2000 μg/ml for *C. albicans *and 31.5 to 250 μg/ml for *C. glabrata*. The culture flasks were incubated with agitation at 35°C. At predetermined time points (0, 0.5, 1, 2, 4, 6, 8, 10, 12, and 24 h following the addition of hydroxychavicol), a 100 μl aliquot was removed from each culture flask and serially diluted in sterile normal saline containing 0.1% polysorbate 80 (Sigma) for the inactivation of hydroxychavicol. A 20 μl aliquot was plated onto a Sabouraud dextrose agar with lecithin and polysorbate 80 (BBL, Becton Dickinson and Company, Cockeysville, MD) plate for colony count determination. When the colony counts were expected to be less than 1000 cfu/ml, samples of 20 μl or 100 μl were taken directly from the test solution and plated or subcultured without dilution. Plates were then incubated at 35°C for 24 to 48 h. The lower limit of accurate and reproducible quantification was 50 cfu/ml for each of the strains.

### Postantifungal effect (PAFE)

The PAFE of hydroxychavicol was performed in RPMI by the method described by Craig and Gudmundsson [21]. *C. albicans *ATCC 90028, *C. tropicalis *ATCC 750, *C. glabrata *ATCC 90030 and *C. parapsilo*sis ATCC 22019 were used as the test strains in this study. One milliliter of the adjusted inoculum suspension (≈ 5 × 10^7 ^cfu/ml) was added to nine ml of RPMI with or without hydroxychavicol, providing the starting inoculum of ≈ 5 × 10^6^cfu/ml. The hydroxychavicol concentrations ranged from one to four times the MIC. After exposures to the hydroxychavicol for 2 h, samples were diluted to 1: 1,000 in prewarmed medium to effectively remove the hydroxychavicol. The diluted cultures were then incubated with agitation (200 rpm) at 35°C and sampling was done after 0, 2, 4, 6, 8, 10, 12, 16 and 24 h for colony counts. The colony counts were determined as described above in time-kill curve studies. The PAFE was calculated by the following equation: PAFE = *T-C*, where *T *represents the time required for the count in the test culture to increase 1 log_10 _cfu/ml above the count observed immediately after drug (hydroxychavicol) removal and *C *represents the time required for the count of the untreated control flask to increase by 1 log_10 _cfu/ml.

### Selection of resistant mutants in vitro

The first step mutant frequency of reference strains of *C. albicans *ATCC 90028, *C. tropicalis *ATCC 750, *C. glabrata *ATCC 90030, *C. parapsilosis *ATCC 22019, *A. flavus *MTCC 2799 and *A. fumigatus *MTCC 1811 were selected, using previously described method [22]. A fungal suspension containing 10^9 ^cfu (100 μl) was plated on SDA containing hydroxychavicol at concentrations equal to two, four and eight times the MIC. Mutation frequency was calculated by counting the total number of colonies appearing after 48 h of incubation at 35°C on the hydroxychavicol containing plate and by dividing the number by the total number of cfu plated.

### Minimum biofilm inhibitory concentrations (MBICs)

The effect of hydroxychavicol on *C. albicans *ATCC 90028 biofilm formation was examined by the microbroth dilution method, similar to MIC assays for planktonic cells [16] as described above. The fungal suspension was prepared from the overnight culture grown in yeast nitrogen base (Difco Laboratories) medium supplemented with 100 mM glucose [23], and the cells were harvested in the late exponential growth phase, washed twice with sterile phosphate-buffered saline (PBS; pH 7.2; Ca^2+ ^and Mg^2+ ^free [Hi Media]) and the turbidity of the suspension was adjusted to 4 McFarland standard (≈ 5 × 10^7 ^cfu/ml). The suspension was diluted in RPMI to obtain ≈ 5 × 10^6 ^cfu/ml as the final inoculums. Twofold serial dilutions of hydroxychavicol were prepared in RPMI in the wells of a 96-well flat-bottom polystyrene microtiter plate (NUNC, Roskilde, Denmark) containing the same media in a volume of 100 μl per well. A 100 μl of above-mentioned suspension was added to each well; the final concentrations of hydroxychavicol ranged from 1.95 to 2000 μg/ml. Amphotericin B (at a final concentration range from 0.0156 to 16 μg/ml) was used as control drug. Following incubation at 35°C for 48 h, absorbance at 490 nm was recorded to assess culture growth. The culture supernatants from each well were then decanted, and planktonic cells were removed by washing the wells with sterile PBS. Biofilm formation was quantified by tetrazolium salt (XTT) reduction assay (see below).

### Minimum biofilm reduction concentrations (MBRCs)

The effect of hydroxychavicol was also examined on preformed *C. albicans *ATCC 90028 biofilm by the method as described previously [24]. Biofilms were prepared by inoculating the wells of a polystyrene microtiter plate in a manner similar to that described above. After incubation at 35°C for 48 h, the culture supernatant from each well was decanted, and the planktonic cells were removed by washing the wells with PBS. Two fold serial dilutions of hydroxychavicol were prepared in RPMI, and 200 μl of each dilution was added to the biofilm in the wells. The plate was further incubated at 35°C for 48 h. The final concentrations of hydroxychavicol ranged from 1.95 to 2000 μg/ml. Amphotericin B (at a final concentration range from 0.0156 to 16 μg/ml) was used as control drug. After the completion of incubation, the plates were decanted and washed three times with 200 μl of sterile PBS to remove loosely adherent cells. Biofilm reduction was quantified by XTT-reduction assay described below.

### XTT-reduction assay

XTT (tetrazolium salt 2, 3-bis (2-methoxy-4-nitro-5-sulfo-phenyl)-2H-tetrazolium-5-carboxanilide) reduction assay was performed by the method as described by Jin et al., [23]. The XTT (Sigma) solution was prepared in PBS (1 mg/ml), filter-sterilized through a 0.22-μm-pore-size filter (Millipore, Bangalore, India) and stored at -80°C until required. Menadione (Sigma) solution (0.4 mM prepared in acetone) was filtered and mixed with XTT solution at a ratio of 1 to 5 by volume before the assay. 200 μl of PBS and 12 μl of the XTT-Menadione solution were added to each of the washed wells and the plate was incubated in the dark for 2 h at 35°C. Following incubation, 100 μl of the solution was transferred to a fresh microtiter plate and, the color change in the solution was measured spectrophometrically using a microtitre plate reader (Multiskan spectrum, Thermo electron, Vantaa, Finland) at 490 nm.

### Propidium iodide uptake assay

The disruptive effect of hydroxychavicol on *Candida albicans *ATCC 90028 cell membranes was assessed by using hydroxychavicol-mediated propidium iodide uptake. One-milliliter volumes of ≈ 5 × 10^7 ^cfu/ml cell suspensions of *C. albicans *in sterile MilliQ water were incubated with two to eight times the MIC (500 to 2000 μg/ml) of hydroxychavicol at 35°C for 60 min under agitation in the dark chamber. Fifteen minutes prior to the completion of incubation, 10 μl each of propidium iodide and sodium deoxycholate solution were added at a final concentration of 25 μg/ml and 2.5 mg/ml "respectively" [14,15]. Amphotericin B at eight times the MIC (4.0 μg/ml) was used as the positive control and, the cells without hydroxychavicol served as the negative (growth) control, treated in similar fashion. After incubation, 50 μl aliquot was transferred into fluorescence-activated cell sorting (FACS) tube (Becton Dickinson Biosciences, CA) containing 950 μl of sterile MilliQ water. Each tube was analyzed using a FACScan flow cytometer (BD-LSR; Becton Dickinson) with Cell Quest Pro software for data acquisition and analysis.

## Results

### Antifungal susceptibility results

The MICs and MFCs of hydroxychavicol were evaluated in vitroagainst 58 strains of yeasts, 39 strains of *Aspergillus *species and 27 strains of dermatophytes and all values are listed in Table 1. Hydroxychavicol exhibited the MICs range between 15.62 to 500 μg/ml for yeasts, 125 to 500 μg/ml for *Aspergillus *species and 7.81 to 62.5 μg/ml for dermatophytes, where as the MFCs were found to be similar or two fold greater than the MICs. Among all the fungal species tested, dermatophytes were found to be the most susceptible species to hydroxychavicol.

**Table 1:** MICs and MFCs of hydroxychavicol for 124 strains of selected fungi determined by the broth microdilution method

Species	No of strains tested	Antifungal activity in μg/ml	
		
		MIC range	MFC range
*C. albicans *ATCC 90028, 10231	2	250	250
*C. albicans *(CI)	23	125 - 500	250 - 500
*C. glabrata *ATCC 90030	1	31.25	31.25
*C. glabrata *(CI)	7	15.62 - 31.25	15.62 - 62.5
*C. krusei *ATCC 6258	1	15.62	15.62
*C. krusei*(CI)	3	15.62 - 31.25	15.62 - 31.25
*C. parapsilosis *ATCC 22019	1	31.25	31.25
*C. parapsilosis *(CI)	5	31.25 - 62.5	31.25 - 62.5
C. *tropicalis *ATCC 750	1	250	250
C. *tropicalis *(CI)	11	125 - 500	250 - 500
*C. neoformans *ATCC 204092	1	62.5	62.5
*C. neoformans *(CI)	2	62.5	62.5
*A*. *flavus *MTCC 1973, 2799	2	250	250
*A*. *flavus *(CI)	11	125 - 500	125 - 500
*A. fumigatus *MTCC 1811	1	250	250
*A. fumigatus *(CI)	17	125 - 500	250 - 500
*A. niger *ATCC 16404	1	125	125
*A. niger *(CI)	6	125 - 250	125 - 250
*A. parasiticus *MTCC 2796	1	250	250
*E. floccosum *MTCC 613	1	15.62	15.62
*E. floccosum*(CI)	1	15.62	31.25
*M. canis *MTCC 2820	1	15.62	31.25
*M. canis *(CI)	3	7.81- 15.62	15.62 - 31.25
*M. gypsium *MTCC 2819	1	15.62	31.25
*M. gypsium*(CI)	2	15.62 - 31.25	31.25 - 62.5
*T. mentagrophytes *ATCC 9533	1	15.62	15.62
*T. mentagrophytes *(CI)	7	15.62 - 31.25	15.62 - 62.5
*T. rubrum *MTCC 296	1	31.25	31.25
*T. rubrum *(CI)	9	15.62 - 62.5	31.25 - 62.5

MIC and MFC of yeast was determined by using higher inoculums [19]. CI, clinical isolate.

### Time kill curve studies

**Figure 2:**
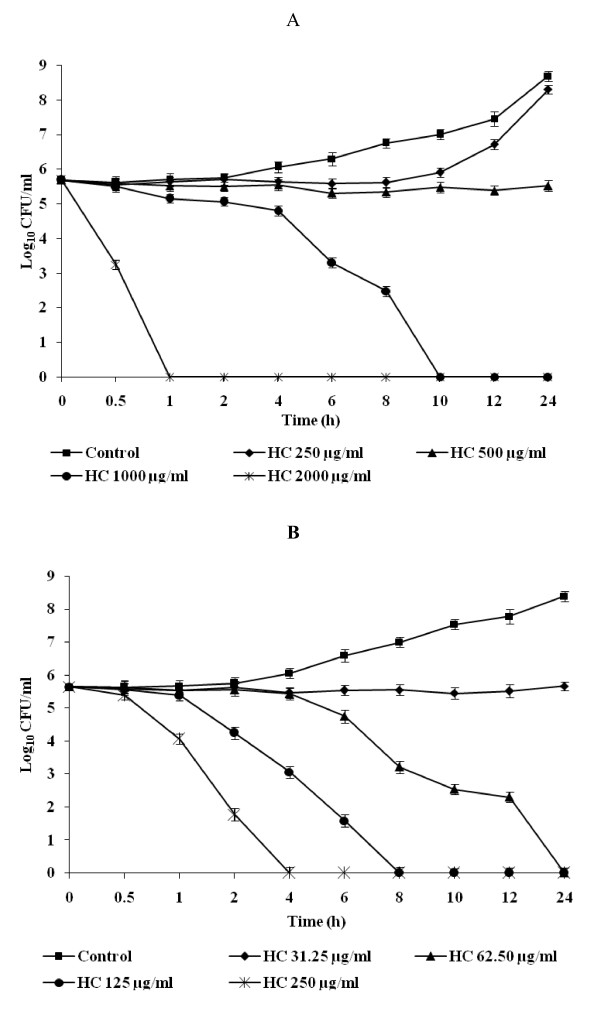
**Time-kill curve plots for *Candida *species following exposure to hydroxychavicol (HC)**. *C. albicans *ATCC 90028 (A), *C. glabrata *ATCC 90030 (B). Each time point represents the mean log_10 _± standard deviations of two different experiments performed in duplicate. *P *values < 0.001 (Student's *t*-test).

The killing activities of hydroxychavicol for *C. albicans *ATCC 90028 and *C. glabrata *ATCC 90030 are shown in Fig. 2. Hydroxychavicol exhibited fungicidal activity against both *Candida *species and the reduction in the number of cfu per milliliter was greater than 3 log units (99.9%). The fungicidal endpoint for *C. albicans *was achieved after 10 and 1 h at 4 × MIC (4 × 250 μg/ml) and 8 × MIC (8 × 250 μg/ml) of hydroxychavicol (Fig. 2A). In C. *glabrata*, killing was observed at a lower concentration of hydroxychavicol due to its lower MIC. There was concentration dependent killing observed in case of C. *glabrata*, with two, four and eight times the MIC exhibited fungicidal activity in 10, 8 and 4 h "respectively".

### PAFE studies

Hydroxychavicol produced significant PAFE against all the *Candida *species tested (Table 2). Increase in the concentration of hydroxychavicol resulted in extended PAFE for all the *Candida spp*. tested. This increase in PAFE was more prominent for *C. albicans *and *C. tropicalis*, where a PAFE of >8 h was exhibited in these organisms at four times the MIC concentration of hydroxychavicol.

**Table 2:** PAFE values of hydroxychavicol for *Candida *species after 2 h of exposure

Species	PAFEs (h) (mean ± SD) at the following multiple of the MIC:		
	
	1 × MIC	2 × MIC	4 × MIC
*C. albicans *ATCC 90028	5.53 ± 0.3	6.34 ± 0.2	8.64 ± 0.3
C. *tropicalis *ATCC 750	4.4 ± 0.6	6.4 ± 0.4	8.70 ± 0.2
*C. glabrata *ATCC 90030	3.08 ± 0.4	3.76 ± 0.6	8.04 ± 0.1
*C. parapsilosis *ATCC 22019	2.0 ± 0.1	4.0 ± 0.2	6.25 ± 0.3

### Frequency of emergence of hydroxychavicol resistant mutants

The frequencies of mutant selection of *C. albicans, C. tropicalis, C. glabrata, C. parapsilosis, A. fumigatus*, and *A. flavus*, are summarized in Table 3. Hydroxychavicol completely suppressed the emergence of mutants at two times its MIC for *A. fumigatus *and *A. flavus*, four times the MIC for *C. albicans *and *C. tropicalis*, and eight times the MIC for *C. glabrata *and *C. parapsilosis *"respectively". This concentration of hydroxychavicol at which no mutant was selected can be defined as the mutation prevention concentration.

**Table 3:** Frequency of mutation with hydroxychavicol

Tested strains	Mutation frequency with hydroxychavicol at:		
	
	2 × MIC	4 × MIC	8 × MIC
*C. albicans *ATCC 90028	2.5 × 10^9^	<10^9^	<10^9^
C. *tropicalis *ATCC 750	2 × 10^9^	<10^9^	<10^9^
*C. glabrata *ATCC 90030	1.5 × 10^9^	1.5 × 10^9^	<10^9^
*C. parapsilosis *ATCC 22019	2 × 10^9^	2 × 10^9^	<10^9^
*A. fumigatus *MTCC 1811	<10^9^	<10^9^	<10^9^
*A. flavus *MTCC 1973	<10^9^	<10^9^	<10^9^

MIC of hydroxychavicol is 31.25 μg/ml for *C. glabrata *and *C. parapsilosis *while as 250 μg/ml for other species tested.

### Biofilm susceptibility assay

**Figure 3:**
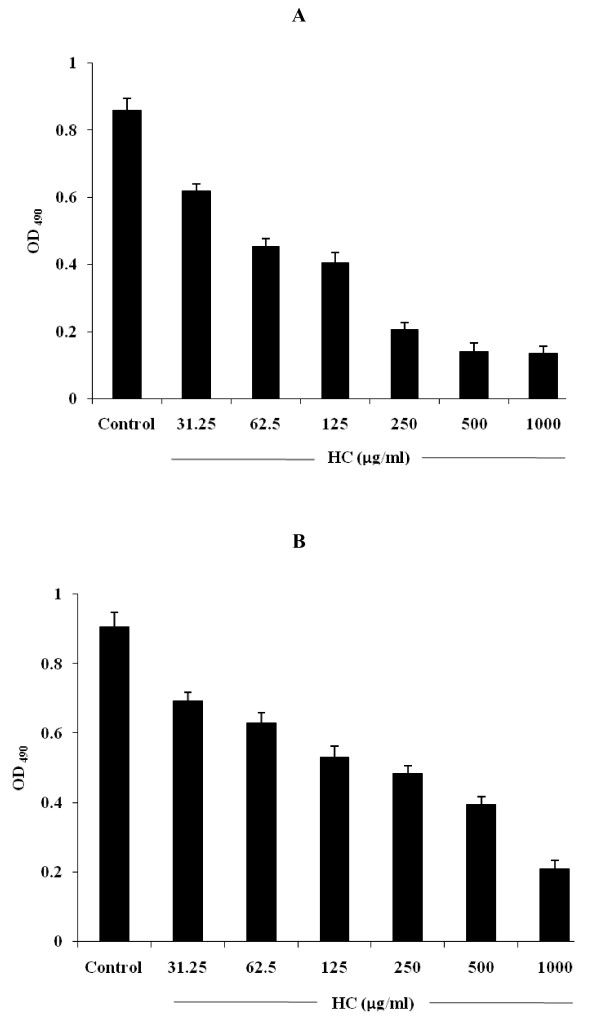
**Inhibitory effect of hydroxychavicol (HC) on the biofilm formation (A) and reduction (preformed) (B) of *C. albicans *ATCC 90028 biofilms**. After incubation, the biofilm viability was quantified by XTT reduction assay at absorbance of 490 nm. The results are expressed as average optical density readings for XTT assays compared to growth control. Values represent the mean and standard deviations of three different experiments performed in quadruplicate. *P *values < 0.05 (Student's *t*-test).

Hydroxychavicol exhibited an inhibitory effect on the biofilm formation and reduction of preformed biofilm of *C. albicans *ATCC 90028. The 50% and 80% biofilm inhibition as well as biofilm reduction are represented in Fig. 3. The MBIC_50 _and MBIC_80 _values of hydroxychavicol were 125 μg/ml and 250 μg/ml, where as the MBRC_50 _and MBRC_80_values were 500 μg/ml and 1000 μg/ml. Reductions of preformed biofilms values were four fold greater than the concentration required to inhibit biofilm formation.

### Effect of hydroxychavicol on membrane permeability

**Figure 4:**
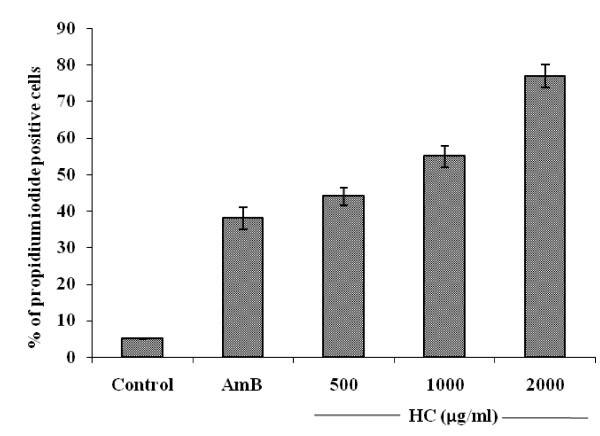
**Uptake of propidium iodide in cell suspension of *C. albicans *ATCC 90028**. Cells (≈ 5 × 10^7^cfu/ml) were exposed to two to eight times (500 to 2000 μg/ml) the MIC of hydroxychavicol (HC) for 60 min. Amphotericin B at eight times the MIC (4.0 μg/ml) was used as the positive control and, the cells without hydroxychavicol served as the growth control. Data represent the mean and standard deviations of two different experiments performed in triplicate. *P *values < 0.05 (Student's *t*-test).

Exposing the cell suspension of *C. albicans *ATCC 90028 to two to eight times (500 to 2000 μg/ml) the MIC of hydroxychavicol for 60 min increased the cell permeability to the fluorescent nucleic acid stain, propidium iodide due to the disruption of membrane integrity. This resulted in the increase in fluorescence in comparison to untreated control (Fig. 4). This increase in fluorescence was proportional to the increase in the hydroxychavicol concentrations.

## Discussion

In this study, we evaluated the antifungal activities of hydroxychavicol against various fungal species. Hydroxychavicol demonstrated fungicidal effects against all the fungal species tested including *Candida *spp., *Aspergillus *spp. and dermatophytes. The fungicidal effect was most pronounced in dermatophytes including *T. rubrum *(MICs and MFCs were 15.62 - 62.5 μg/ml) which is the etiological agent of 80 to 93% of all clinical infections produced by dermatophytes [3]. Hydroxychavicol also exhibited concentration dependent killing and extended PAFE of > 8 h. In the concentration range of 250-1000 μg/ml it completely suppressed the emergence of mutants of various *Candida *and *Aspergillus *species tested.

*C. albicans *is most commonly associated with biofilm formation, and the increase in *Candida *infections in the last decades has almost paralleled the increase and widespread use of a broad range of medical implant devices (such as stents, prostheses, implants, endotracheal tubes, pacemakers, and catheters), mainly in populations with impaired host defenses. Biofilm formation on medical devices can negatively impact the host by causing the failure of the device and by serving as a reservoir or source for future continuing infections [25]. Hydroxychavicol was effective in inhibiting the *C. albicans *generated biofilm with 80% inhibition of biofilm was observed at the MIC concentration (250 μg/ml). However the reduction of the preformed biofilm was seen at four fold greater concentrations.

There have been reports on the antifungal activities of *P. betle*. Pongpech and Prasertsilpe [26] found that *P. betle *gel inhibited growth of dermatophytes that cause ringworm and growth of *Candida *species more effectively than tolnaftate and with a similar inhibitory effect to that of clotrimazole. Recently, Trakranrungsie et al [27] also reported the antidermatophytic activity of *P. betle *extract against *M. canis, M. gypseum and T. mentagrophyte *by broth dilution method and showed that *P. betle *exhibited more effective antifungal properties with average IC_50 _and IC_90 _values ranging from 110.44 to 119.00 μg/ml and 230.40 to 492.30 μg/ml "respectively".

Hydroxychavicol is one of the major constituents of *P. betle*. It has been extensively reported for its antibacterial activity [12,13]. However its antifungal activity has not been reported so far. Here in this study we have for the first time reported the antifungal potential of hydroxychavicol.

Propidium iodide is a fluorescent nucleic acid stain that is unable to penetrate the cell membrane structures of healthy cells. However, cells with damaged or permeabilised cell membranes do not exclude propidium iodide. Therefore, propidium iodide staining of cells indicates cytoplasmic membrane (bacteria) and plasma membrane (yeast) damage [28]. Sodium deoxycholate was used in this study as it is reported to enhance the diffusion of propidium iodide across the cell wall to pass through the damaged yeast cell membranes [29,15]. Interestingly, the growth controls did not show dye uptake in the presence of deoxycholate as the deoxycholate is nontoxic to *C. albicans *[29]. The increased uptake of propidium iodide in the hydroxychavicol treated cells of *C. albicans *in our study, further confirmed the earlier findings that hydroxychavicol alters the cell membrane structure, resulting in the disruption of the permeability barrier of microbial membrane structures [30].

The clinical applications of hydroxychavicol were challenging to interpret in this study due to a lack of pharmacokinetic and safety studies. However its comparable cytotoxicity profile with that of thymol widely used natural phenolic as food preservative and oral care agent in the earlier study [12] is indicative of the safety of this compound.

## Conclusions

The results presented in this study are the first information of hydroxychavicol for antifungal activity. Hydroxychavicol exhibited a broad range antifungal activity against clinically significant human fungal species. Further studies are therefore warranted in order to explore of this natural compound for topical use in fungal infections particularly dermatomycoses.

## Abbreviations

MIC: minimum inhibitory concentration; MFC: minimum fungicidal concentration; ATCC: american type culture collection; MTCC: microbial type culture collection; cfu: colony forming unit; MOPS: morpholinepropanesulfonic acid; DMSO: dimethyl sulfoxide; PAFE: postantifungal effect; MBIC: minimum biofilm inhibitory concentration; MBRC: minimum biofilm reduction concentration; XTT: 2, 3-bis (2-methoxy-4-nitro-5-sulfo-phenyl)-2H-tetrazolium-5-carboxanilide.

## Competing interests

The authors declare that they have no competing interests.

## Authors' contributions

IA was carried out all experimental work, data acquisition and analysis, literature search and writing the manuscript. IAK and FA were responsible for study concept, designing and coordinating the research, supervising the work and revising the manuscript. FGK is the collaborator from the Medical College and provided the clinical inputs in the manuscript. KAS, BDG, NKS and PD involved in extraction and characterization of hydroxychavicol from *Piper betle*. GNQ was involved in critical evaluation of the manuscript. All authors have read and approved the final manuscript.
